# Inhibition of the alternative complement activation pathway in traumatic brain injury by a monoclonal anti-factor B antibody: a randomized placebo-controlled study in mice

**DOI:** 10.1186/1742-2094-4-13

**Published:** 2007-05-02

**Authors:** Iris Leinhase, Michal Rozanski, Denise Harhausen, Joshua M Thurman, Oliver I Schmidt, Amir M Hossini, Mohy E Taha, Daniel Rittirsch, Peter A Ward, V Michael Holers, Wolfgang Ertel, Philip F Stahel

**Affiliations:** 1 Department of Trauma and Reconstructive Surgery, Charité University Medical School, Campus Benjamin Franklin, 12200 Berlin, Germany; 2 Departments of Medicine and Immunology, University of Colorado Health Sciences Center, Denver, CO 80262, USA; 3 Department of Pathology, University of Michigan Medical School, Ann Arbor, MI 48109, USA; 4 Department of Orthopedic Surgery, Denver Health Medical Center, University of Colorado School of Medicine, Denver, CO 80204, USA

## Abstract

**Background:**

The posttraumatic response to traumatic brain injury (TBI) is characterized, in part, by activation of the innate immune response, including the complement system. We have recently shown that mice devoid of a functional alternative pathway of complement activation (factor B-/- mice) are protected from complement-mediated neuroinflammation and neuropathology after TBI. In the present study, we extrapolated this knowledge from studies in genetically engineered mice to a pharmacological approach using a monoclonal anti-factor B antibody. This neutralizing antibody represents a specific and potent inhibitor of the alternative complement pathway in mice.

**Methods:**

A focal trauma was applied to the left hemisphere of C57BL/6 mice (*n *= 89) using a standardized electric weight-drop model. Animals were randomly assigned to two treatment groups: (1) Systemic injection of 1 mg monoclonal anti-factor B antibody (*mAb 1379*) in 400 μl phosphate-buffered saline (PBS) at 1 hour and 24 hours after trauma; (2) Systemic injection of vehicle only (400 μl PBS), as placebo control, at identical time-points after trauma. Sham-operated and untreated mice served as additional negative controls. Evaluation of neurological scores and analysis of brain tissue specimens and serum samples was performed at defined time-points for up to 1 week. Complement activation in serum was assessed by zymosan assay and by murine C5a ELISA. Brain samples were analyzed by immunohistochemistry, terminal deoxynucleotidyl transferase dUTP nick-end labeling (TUNEL) histochemistry, and real-time RT-PCR.

**Results:**

The *mAb 1379 *leads to a significant inhibition of alternative pathway complement activity and to significantly attenuated C5a levels in serum, as compared to head-injured placebo-treated control mice. TBI induced histomorphological signs of neuroinflammation and neuronal apoptosis in the injured brain hemisphere of placebo-treated control mice for up to 7 days. In contrast, the systemic administration of an inhibitory anti-factor B antibody led to a substantial attenuation of cerebral tissue damage and neuronal cell death. In addition, the posttraumatic administration of the *mAb 1379 *induced a neuroprotective pattern of intracerebral gene expression.

**Conclusion:**

Inhibition of the alternative complement pathway by posttraumatic administration of a neutralizing anti-factor B antibody appears to represent a new promising avenue for pharmacological attenuation of the complement-mediated neuroinflammatory response after head injury.

## Background

**Figure 1:**
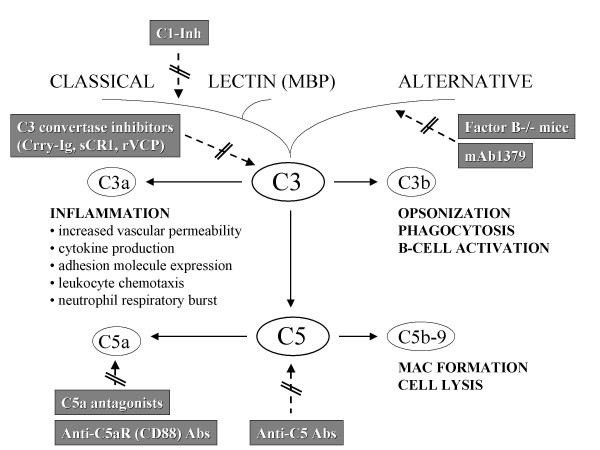
**Schematic drawing of complement activation pathways, immunological functions, and specific inhibitory strategies used in experimental head injury models**. Complement is activated either through the classical, lectin, or alternative pathways. Activation of complement leads to the formation of multi-molecular enzyme complexes termed convertases that cleave C3 and C5, the central proteins of the complement system. The proteolytic fragments generated by cleavage of C3 and C5 mediate most of the biological activities of complement. C3b, and proteolytic fragments generated from C3b, are important opsonins that target pathogens for removal by phagocytic cells via complement receptors specific for these proteins. These molecules have furthermore been shown to bridge innate to adaptive immune responses by the activation of B-cells. C3a and C5a are potent anaphylatoxins with chemotactic and inflammatory properties. Generation of C5b by cleavage of C5 initiates the formation of the membrane attack complex (MAC, C5b-9) through the terminal complement pathway. The MAC forms through the self-association of C5b along with C6 through C9 and leads to the formation of a large membranolytic complex capable of lysing cells. Therapeutic modalities from experimental head injury models are aimed either at blocking specific activation pathways (classical, alternative), components (C5) and proteolytic fragments (C5a, C5aR), or by a "pan"-inhibition of C3 convertases, leading to a complete shut-down of complement activation. *See text for references and explanations*. C1-Inh, C1-inhibitor; C5aR, anaphylatoxin C5a receptor (CD88); Crry-Ig, Complement receptor type 1-related protein y, IgG1-linked murine recombinant fusion protein; MBP, mannose-binding protein; rVCP, recombinant Vaccinia virus complement control protein; sCR1, soluble complement receptor type 1.

Traumatic brain injury (TBI) represents a neuroinflammatory disease which is in large part mediated by an early activation of the innate immune system [1-4]. In this regard, the complement system has been identified as an important early mediator of posttraumatic neuroinflammation [5-7]. Research strategies to prevent the neuroinflammatory pathological sequelae of TBI have largely failed in translation to clinical treatment [8-14]. This notion is exemplified by the recent failure of the "CRASH" trial (Corticosteroid randomization after significant head injury). This large-scale multicenter, placebo-controlled randomized study was designed to assess the effect of attenuating the neuroinflammatory response after TBI by administration of high-dose methylprednisolone [15]. The trial was unexpectedly aborted after enrollment of 10,008 patients based on the finding of a significantly increased mortality in the steroid cohort, compared to the placebo control group [15]. These data imply that the "pan"-inhibition of the immune response by the use of glucocorticoids represents a too broad and unspecific approach for controlling neuroinflammation after TBI [16]. Thus, research efforts are currently focusing on more specific and sophisticated therapeutic modalities, such as the inhibition of the complement cascade [17-19]. Several complement inhibitors have been investigated in experimental TBI models [20-26]. However, most modalities of complement inhibition have focussed on interfering with the cascade at the central level of the C3 convertases, where the three activation pathways merge (Fig. 1) [20,21,25-27]. Other approaches were designed to inhibit the main inflammatory mediators of the complement cascade, such as the anaphylatoxin C5a [22,28-30]. Only more recently, increased attention was drawn to the "key" role of the alternative pathway in the pathophysiology of different inflammatory conditions outside the central nervous system (CNS) [31-34]. We have recently reported that factor B knockout (*fB-/-*) mice, which are devoid of a functional alternative pathway, show a significant neuroprotection after TBI, compared to head-injured wild-type mice [35]. These data served as a baseline for the present study, where we extrapolated the positive findings in the knockout mice to a pharmacological approach. We therefore used a neutralizing monoclonal anti-factor B antibody which was recently described as a highly potent inhibitor of the alternative pathway in mice [31,34,36,37] in the setting of a standardized model of closed head injury [38].

## Methods

### Animals

All experiments were performed in adult male mice of the C57BL/6 strain (*n *= 89 in total) purchased from Jackson Laboratory (Bar Harbor, ME). The mice were bred in a selective pathogen-free (SPF) environment and under standardized conditions of temperature (21°C), humidity (60%), light and dark cycles (12:12 h), with food and water provided *ad libitum*. Experiments were performed in compliance with the standards of the *Federation of European Laboratory Animal Science Association *(FELASA) and were approved by the institutional animal care committee (*Landesamt für Arbeitsschutz, Gesundheitsschutz und technische Sicherheit Berlin*, Berlin, Germany, No. G0099/03).

### Trauma model

Mice were subjected to experimental TBI using a standardized weight-drop device, as previously described [26,35,39,40]. In brief, after induction of isoflurane anesthesia, the skull was exposed by a midline longitudinal scalp incision. The head was fixed and a 250 g weight was dropped on the skull from a height of 2 cm, resulting in a focal blunt injury to the left hemisphere. After trauma, the mice received supporting oxygenation with 100% O_2 _until fully awake. The extent of posttraumatic neurological impairment was assessed at defined time intervals after trauma (*t *= 1 h, 4 h, 24 h, and 7 days) using a standardized *Neurological Severity Score *(NSS), as described below.

### Treatment protocol

The inhibitory monoclonal anti-factor B antibody (*mAb 1379*) used in this study was previously described and the selected dosage was in the titrated range used in other studies on murine models of inflammation [34,36,37]. The antibody itself does not have any complement-activating properties. Mice were randomly assigned to two treatment groups: (1) Systemic injection of 1 mg *mAb 1379 *in 400 μl phosphate-buffered saline (PBS) at 1 hour and 24 hours after trauma; (2) Systemic injection of vehicle only (400 μl PBS), as placebo control, at identical time-points after trauma. Concealed allocation to the two treatment cohorts was performed after assessment of the baseline NSS at 1 hour after trauma, in order to ensure equal injury severity between the groups. The systemic (i.p.) route of administration and the time window of injection were selected based on the breakdown of the blood-brain barrier (BBB) for up to 24 hours after trauma [38,41]. This allows a "time window" for peripherally administered compounds to reach the intrathecal compartment and exert pharmacological effects in the CNS [26,39,40,42]. Furthermore, the systemic injection early after trauma represents an approach with potential clinical implications. In order to induce a continuing complement inhibition during the acute inflammatory phase in the first days, injections were repeated at 24 hours.

Subgroups of mice (*n *= 10 per group and time-point) were euthanized by isoflurane anesthesia and decapitated at *t *= 4 h, 24 h, and 7 days. Brains were immediately extracted, snap-frozen in liquid nitrogen and stored at -80°C until analysis by immunohistochemistry, TUNEL histochemistry and real-time RT-PCR. In addition, serum samples were collected at identical time-points for determination of complement activation levels. Sham-operated and untreated normal mice served as negative controls.

### Neurological Severity Score (NSS)

A previously characterized 10-parameter score was used for assessment of posttraumatic neurological impairment, as described elsewhere in detail [41,43]. The NSS was assessed in a blinded fashion by two different investigators at the time-points *t *= 1 h, 4 h, 24 h, and 7 days after trauma. The score comprises 10 individual parameters, including tasks on motor function, alertness, and physiological behavior, whereby one point is given for failure of the task, and no point for succeeding. A maximum NSS score of 10 points indicates severe neurological dysfunction, with failure of all tasks.

### Mouse C5a ELISA

Serum levels of the complement anaphylatoxin C5a were determined by a mouse-specific ELISA developed in the laboratory of Dr. P.A. Ward (Ann Arbor, MI), as previously described [35,44]. In brief, ELISA plates (Immulon 4HBX, Thermo Labsystems, Milford, MA) were coated with 5 μg/ml of purified monoclonal anti-mouse C5a IgG (BD Pharmingen, San Diego, CA). After blocking of non-specific binding sites with 1% milk (Roth, Karlsruhe, Germany) in PBS (Gibco-Invitrogen, Carlsbad, CA) containing 0.05% TWEEN 20 (Sigma-Aldrich), the plate was coated with 100 μl of each serum diluted 1:20 (in 0.1% milk in PBS containing 0.05% TWEEN) and murine recombinant mouse C5a at defined concentrations for establishing the standard curve. After incubation and subsequent washing steps, biotinylated monoclonal anti-mouse C5a antibody was added at 500 ng/ml (BD Pharmingen) followed by washing steps and incubation with streptavidin-peroxidase at 400 ng/ml (Sigma-Aldrich).

For colorimetric reaction, 0.4 mg/ml *o-*phenylenediamine dihydrochloride with 0.4 mg/ml urea hydrogen peroxide in 0.05 M phosphate citrate buffer (Sigma-Aldrich) was added and the color reaction was stopped with 3 M sulfuric acid. Absorbance was read at 490 nm using a "SpectraMax 190" reader (Molecular Devices, Sunnyvale, CA). All samples were analyzed in duplicate and results were calculated from the means of duplicate sample analysis. The standard curve was linear from 0.1 ng/ml to 50 ng/ml.

### Quantification of alternative pathway complement activity

Alternative pathway complement activity in mouse serum was quantified as previously described [26,36]. Briefly, at the above-mentioned defined time-points, whole blood was collected and spun down, serum was aliquoted and stored at -80°C until analyzed. Ten microlitres of serum from each animal was incubated with 10^9 ^zymosan particles (Sigma-Aldrich, St. Louis, MO) at 37°C for 30 min in a master mix containing final concentrations of 5 mM MgCl_2 _and 10 mM EGTA and brought up to 100 μl in calcium-free PBS. C3 deposition on the particles was detected with a FITC-labeled antibody to C3 (Cappel, Durham, USA) diluted 1:100 and fluorescence was measured by flow cytometry. Complement activity was calculated using the formula:

100×(sample mean channel fluorescence−background [no serum])(positive control mean channel fluorescence−background)
 MathType@MTEF@5@5@+=feaafiart1ev1aaatCvAUfKttLearuWrP9MDH5MBPbIqV92AaeXatLxBI9gBaebbnrfifHhDYfgasaacH8akY=wiFfYdH8Gipec8Eeeu0xXdbba9frFj0=OqFfea0dXdd9vqai=hGuQ8kuc9pgc9s8qqaq=dirpe0xb9q8qiLsFr0=vr0=vr0dc8meaabaqaciaacaGaaeqabaqabeGadaaakeaacqaIXaqmcqaIWaamcqaIWaamcqGHxdaTdaWcaaqaaiabcIcaOiabdohaZjabdggaHjabd2gaTjabdchaWjabdYgaSjabdwgaLjabbccaGiabd2gaTjabdwgaLjabdggaHjabd6gaUjabbccaGiabdogaJjabdIgaOjabdggaHjabd6gaUjabd6gaUjabdwgaLjabdYgaSjabbccaGiabdAgaMjabdYgaSjabdwha1jabd+gaVjabdkhaYjabdwgaLjabdohaZjabdogaJjabdwgaLjabd6gaUjabdogaJjabdwgaLjabgkHiTiabdkgaIjabdggaHjabdogaJjabdUgaRjabdEgaNjabdkhaYjabd+gaVjabdwha1jabd6gaUjabdsgaKjabbccaGiabcUfaBjabd6gaUjabd+gaVjabbccaGiabdohaZjabdwgaLjabdkhaYjabdwha1jabd2gaTjabc2faDjabcMcaPaqaaiabcIcaOiabdchaWjabd+gaVjabdohaZjabdMgaPjabdsha0jabdMgaPjabdAha2jabdwgaLjabbccaGiabdogaJjabd+gaVjabd6gaUjabdsha0jabdkhaYjabd+gaVjabdYgaSjabbccaGiabd2gaTjabdwgaLjabdggaHjabd6gaUjabbccaGiabdogaJjabdIgaOjabdggaHjabd6gaUjabd6gaUjabdwgaLjabdYgaSjabbccaGiabdAgaMjabdYgaSjabdwha1jabd+gaVjabdkhaYjabdwgaLjabdohaZjabdogaJjabdwgaLjabd6gaUjabdogaJjabdwgaLjabgkHiTiabdkgaIjabdggaHjabdogaJjabdUgaRjabdEgaNjabdkhaYjabd+gaVjabdwha1jabd6gaUjabdsgaKjabcMcaPaaaaaa@C108@

### Immunohistochemistry

Immunohistochemical stainings of serial coronal cryosections (8 μm) of brain tissue were performed using a biotin/avidin/peroxidase technique with diaminobenzidine tetrahydrochloride as chromogen (Vector, Burlingame, CA). The following primary antibodies were used as cell-markers: monoclonal anti-NeuN for neurons (1:2,000; Chemicon, Hampshire, UK); polyclonal rabbit anti-GFAP for astrocytes (1:100; Shandon Immunon, Pittsburgh, PA) and monoclonal rat anti-CD11b for microglia and monocytes/macrophages (1:100; Accurate Chemical, Westbury, NY). For negative control, non-immunized IgG (Vector) was used at equal dilutions.

### TUNEL assay

The terminal deoxynucleotidyl transferase dUTP nick-end labeling (TUNEL) technique was applied to determine the extent of neuronal cell death in tissue sections. Herefore, the commercially available ''Fluorescein *In Situ *Cell Death Detection Kit'' (Roche Diagnostics GmbH, Mannheim, Germany) was used according to the manufacturer's instructions, as previously described [35]. In brief, slides were dried for 30 min followed by fixation in 10% formalin solution at RT. After washing in PBS, sections were incubated in ice-cold ethanol-acetic acid solution (3:1), washed in PBS and incubated with 3% Triton X-100 solution for 60 min at RT for permeabilization. Slides were then incubated with the TdT-enzyme in reaction buffer containing fluorescein-dUTP for 90 min at 37°C. Negative control was performed using only the reaction buffer without TdT enzyme. Positive controls were performed by digesting with 500 U/ml DNase grade I solution (Roche). To preserve cells for comparison, slices were covered with Vectashield^® ^mounting medium containing 4',6'-diamino-2-phenylindole (DAPI; Vector). All samples were evaluated immediately after staining using an ''Axioskop 40'' fluorescence microscope (Zeiss, Germany) at 460 nm for DAPI and 520 nm for TUNEL fluorescence. Data were analyzed by Alpha digi doc 1201 software (Alpha Innotech, San Leandro, CA).

### Real-time RT-PCR

Changes in the expression profiles of pro- and anti-apoptotic as well as complement-regulatory genes were determined by semi-quantitative two-step real-time RT-PCR using commercially available and custom-made murine-specific primers shown in table 1. This technique was previously described [26]. In brief, brains were homogenized per hemisphere in Qiazol^® ^buffer (Qiagen, Hilden, Germany). RNA was isolated and further purified using RNeasy^® ^Mini-kits (Qiagen) and RNA concentrations were measured using a spectrophotometer (Bio-Rad, Munich, Germany). From each brain hemisphere, 2 μg RNA were reversed transcribed using random nonamer and oligo-dT16mer primers (Operon Biotechnologies, Cologne, Germany) with Omniscript^® ^kits (Qiagen), according to the manufacturer's instructions. Real-time RT-PCR was performed using validated commercially available and custom designed primer-probe^® ^sets (Qiagen) and optimized protocols on the Opticon^® ^real-time PCR Detection System (Bio-Rad). For quantification of gene expression levels, GAPDH amplicons were generated and used as a house-keeping internal control gene. Relative gene expression levels were calculated in relation to the corresponding GAPDH gene expression levels.

**Table 1:** Murine primer sequences used for real-time RT-PCR analysis of intracerebral gene expression

	Gene ID at NCBI *	**GeneBank Accession No.**	**Length of amplicons**	**Primer sequence**	**Probe Sequence**	**Order No. Qiagen**
**GAPDH**^#^	14433	NM_008084	136 bp	commercially available Genexpression Assay QuantiTect Mm_GAPD		241012
**Bcl-2**	12043	NM_009741 NM_177410	118 bp	commercially available Genexpression Assay QuantiTect Mm_Bcl-2		241118
**Fas**	14102	NM_007987	96 bp	commercially available Genexpression Assay QuantiTect Mm_ Tnfsf6		241122
**C1-Inh**	12258	NM_009776	134 bp	AACTTAGAACTCATCAACACCT GTTATCTTCCACTTGGCACTC	ACACCTGCCTCGTCCT	custom made

* NCBI, National Center for Biotechnology Information

^# ^Housekeeping gene

### Statistical analysis

Statistical analysis was performed using commercially available software (SPSS 9.0 for Windows™). Differences in serum complement activity levels and in intracerebral gene expression levels between the groups were determined by the unpaired Student's *t*-test. The repeated measures analysis of variance (ANOVA) was used for assessing differences in neurological scores (NSS). A *P*-value < 0.05 was considered statistically significant.

## Results

### mAb 1379 inhibits complement activation after TBI

**Figure 2:**
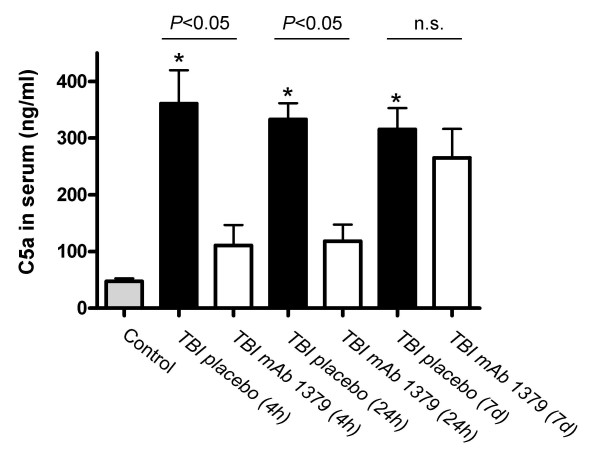
**Posttraumatic injection of *mAb 1379 *attenuates C5a levels in serum of head-injured mice**. Serum samples from mice treated with placebo or *mAb1379 *were analyzed by a specific murine C5a ELISA, as described in the methods section. C5a levels were significantly decreased in *mAb1379*-treated mice compared to placebo controls at 4 and 24 hours (*P *< 0.05, unpaired Student's *t*-test), but not at 7 days after trauma (n.s., not significant). Data are shown as mean levels ± SD of *n *= 3 animals per group and time-point. *P < 0.05 head-injured placebo-injected mice *vs*. normal controls (unpaired Student's *t*-test). TBI, traumatic brain injury.

**Figure 3:**
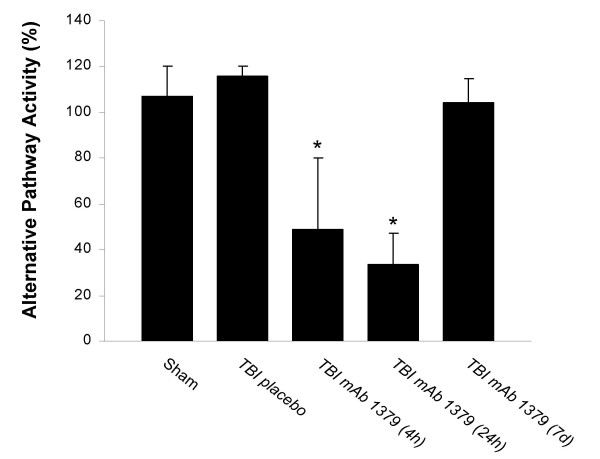
**Functional assessment of *mAb 1379 *on alternative complement pathway activity after traumatic brain injury (TBI)**. Relative alternative pathway complement activity levels (normalized to 100%) were determined by zymosan assay in murine serum samples, as described in the methods section. The *mAb 1379*-injected mice showed a significant decrease in alternative complement activity compared to placebo-injected mice at 4 hours and 24 hours, but not at 7 days after TBI. Data are shown as mean levels ± SD of *n *= 5 animals per group and time-point. *P < 0.05, for TBI_*mAb 1379 *(4 h) and TBI_*mAb 1379 *(24 h) vs. TBI_placebo and sham; unpaired Student's *t*-test.

The induction of TBI lead to a significant extent of systemic complement activation within 4 hours after trauma, as revealed by significantly increased anaphylatoxin C5a serum levels (*P *< 0.05 vs. control, unpaired Student's *t*-test; Fig. 2). Peak C5a levels at 4 h after head injury were as high as 450 ng/ml, compared to 42–53 ng/ml in controls. C5a levels in serum remained significantly elevated for up to 7 days after head injury (Fig. 2). In contrast, the systemic (i.p.) injection of 1 mg *mAb 1379 *at one hour post trauma lead to a significant reduction of anaphylatoxin C5a levels in serum at 4 h and 24 h after head injury. The mean C5a levels (± SD) were reduced from 361 ± 59 ng/ml (TBI 4 h) and 333 ± 29 ng/ml (TBI 24 h) in the placebo group to 111 ± 36 ng/ml (TBI 4 h) and 118 ± 30 ng/ml (TBI 24 h) in the *mAb 1379 *group (*P *< 0.05, unpaired Student's *t*-test; Fig. 2). However, a repeated injection of *mAb 1379 *at 24 hours did not mediate a prolonged inhibition of C5a levels for up to 7 days after trauma (316 ± 37 ng/ml in the placebo group *vs*. 265 ± 51 ng/ml in the *mAb 1379 *group, *P *> 0.05; Fig. 2). Similarly to the reduced C5a levels, the *mAb 1379 *led to a significant reduction of alternative pathway complement activity in serum at 4 h and 24 h after TBI, as assessed by zymosan assay (*P *< 0.05 *vs*. PBS-injected TBI mice, unpaired Student's *t*-test; Fig. 3). The repeated injection of *mAb 1379 *at 24 hours could not maintain the alternative pathway inhibition for up to 7 days after trauma (*P *> 0.05 *vs*. PBS-injected TBI mice; Fig. 3).

### Clinical outcome

**Figure 4:**
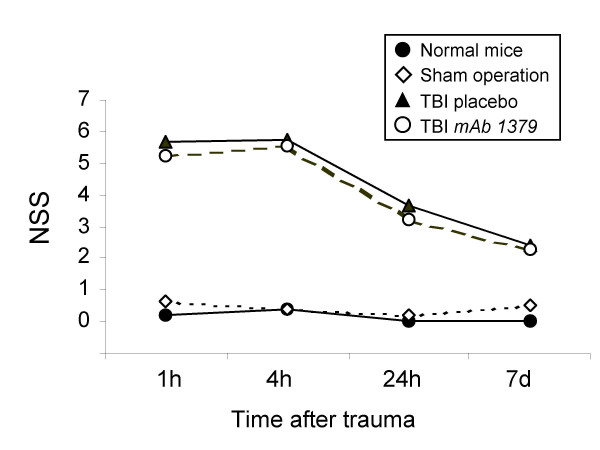
**Neurological outcome after head injury is not altered by injection of *mAb 1379***. The extent of neurological impairment was assessed using a standardized 10-parameter *"Neurological Severity Score" *(NSS) in normal, sham-operated, and head-injured mice from 1 hour to 7 days after trauma (total: *n *= 89 mice). Neurological assessment was performed by two investigators in a blinded fashion. A maximal score of 10 points corresponds to a severe neurological impairment, while a score of 0 points reflects normal behavior [41,43]. The graph shows median levels of the groups at different time-points. No statistically significant differences where found at any time-point between head-injured mice treated with either *mAb 1379 *or placebo (*P *> 0.05, repeated measures ANOVA). TBI, traumatic brain injury.

Evaluation of neurological tasks was performed by two investigators who were blinded about the treatment groups. The mortality from brain injury in this model was below 10% within 7 days, as previously reported [41]. No difference in mortality was observed between head-injured mice in the placebo vs. the *mAb 1379 *injected group (data not shown). With regard to the neurological outcome, the 'nil' and sham control mice showed a normal behavior, as reflected by low mean NSS scores of 0 to 0.67 points (range:0–2 points). In contrast, head-injured mice in both treatment groups had a significantly increased NSS at all time-points assessed for up to 7 days after trauma, compared to the control groups (*P *< 0.05, repeated measures ANOVA; Fig. 4). No significant differences in neurological scores were observed between the groups treated with vehicle *vs*. the *mAb 1379*, as shown in Fig. 4. A spontaneous neurological recovery was seen in both treatment groups over time, as reflected by a decreased NSS at 7 days (vehicle: 2.40 ± 0.52, mean ± SD; *mAb 1379*: 2.30 ± 0.30) compared to 1 hour after TBI (vehicle: 5.67 ± 0.33; *mAb 1379*: 5.27 ± 0.31).

**Figure 5:**
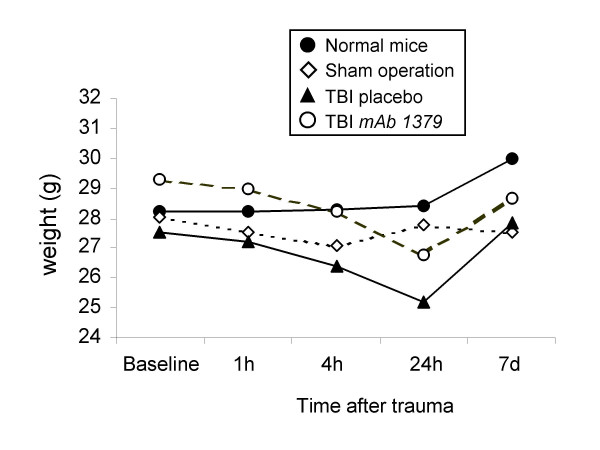
**Kinetics of body weight changes for up to 7 days after traumatic brain injury (TBI)**. Both TBI groups had a decrease in body weight at 24 hours after trauma, compared to baseline values. No significant changes were seen between the *mAb 1379*- *vs*. placebo-injected groups (*P *> 0.05, repeated measures ANOVA). Head-injured mice recovered their baseline body weight by 7 days. Median values are shown for a total of *n *= 89 mice.

Both treatment groups had a weight loss of approximately 10% of their initial body weight within 24 h after trauma, and regained their baseline values by 7 days. No significant differences in body weight were observed between the vehicle and *mAb 1379 *groups (Fig. 5).

### Histomorphological outcome

**Figure 6:**
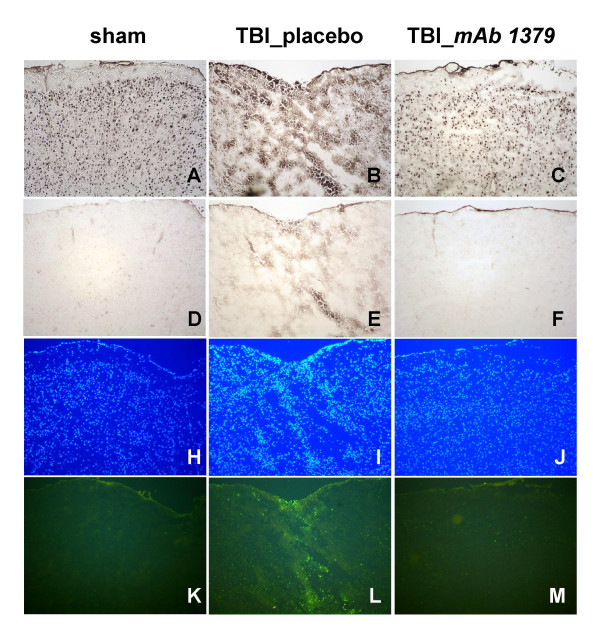
**Neuroprotection at the brain tissue level by *mAb1379 *treatment of head-injured mice**. Adjacent cryosections of 6–8 μm thickness are shown for sham-operated (**panels A, D, H, K**) and head-injured mice treated by placebo (**panels B, E, I, L**) or *mAb 1379 *(**panels C, F, J, M**) at the time-point of 7 days. Immunohistochemical staining by the use of a neuron-specific marker (NeuN, **panels A-C**) shows a significant tissue destruction of the cortical neuronal layers of the injured hemisphere in placebo-injected mice (**B**). In contrast, the injured hemisphere appears largely protected in *mAb 1379*-treated mice (**C**), showing a similarly intact neuronal cell layer morphology as in sham-operated animals (**A**). The staining of infiltrating leukocytes by a marker for complement receptor type 3 (CD11b, **panels D-F**) shows positive cells in the disrupted subarachnoid space of placebo-injected mice (**E**), but not in mAb 1379-injected animals (**F**). Furthermore, TUNEL-histochemistry (**panels K-M**) revealed positive cells in the injured hemisphere of placebo-injected head-injured mice (**L**), but not in mAb 1379-treated animals (**M**). The 4',6'-diamino-2-phenylindole (DAPI) stainings (**panels H-J**) show the overall nuclear morphology in adjacent sections to those stained by TUNEL. TBI, traumatic brain injury. Original magnifications: 100 ×.

Morphologically, the placebo-injected mice exhibited a massive destruction of their cortical neuronal layers for up to 7 days after trauma, as determined by immunohistochemistry using a specific anti-NeuN Ab as a neuronal marker (Fig. 6B). In contrast, the *mAb 1379*-injected mice showed signs of neuronal protection and restoration of the cortical layers to a similar anatomy as in sham-operated mice (Fig. 6A,C). A similar extent of neuroprotection in *mAb 1379*-treated mice was seen in the CA3/CA4 sublayers of the hippocampus, compared to placebo-treated mice (data not shown). The staining of brain tissue section using an anti-CD11b Ab revealed infiltration of CD11b-positive inflammatory cells at the contusion site of brain-injured mice in the placebo group, but not in the *mAb 1379 *group (Fig. 6E,F).

The assessment of intracerebral cell death by TUNEL histochemistry revealed a dramatic increase in TUNEL-positive neurons in the injured left hemispheres of PBS-injected mice at 4 hours after trauma, as previously described for this TBI model [35]. TUNEL-positive cells were detected within the contused area (Fig. 6L) and the hippocampus (not shown) of the injured hemisphere for for up to 7 days after trauma, as compared to sham-operated animals (Fig. 6K). In contrast, the *mAb 1379 *treated mice showed a clearly attenuated extent of intracerebral cell death in the ipsilateral hippocampus (not shown) and cortex around the contusion zone for up to 7 days after trauma (Fig. 6M). Immunohistochemical staining of adjacent sections to those analyzed by TUNEL histochemistry by cell markers for neurons (anti-NeuN), astrocytes (anti-GFAP), and microglia and infiltrating leukocytes (anti-CD11b), revealed that neurons were the predominant TUNEL-positive cell type in all sections taken from the injured hemisphere in PBS-treated mice. Neurons were also confirmed as the predominant TUNEL-positive cell-type by their typical cellular size, morphology, and position in typical neuronal layers. In addition, some infiltrating leukocytes within the contusion site were shown to be TUNEL-positive at the time-point of 7 days after trauma (Fig. 6L). TUNEL-positive cells and the extent of cortical tissue destruction were less apparent in the contralateral (right) hemisphere as compared to the injured (left) hemisphere at all time-points assessed after trauma (data not shown). The representative microphotographs shown in Fig. 6 were highly reproducible in all tissue sections and animals assessed.

### Intracerebral gene regulation

**Figure 7:**
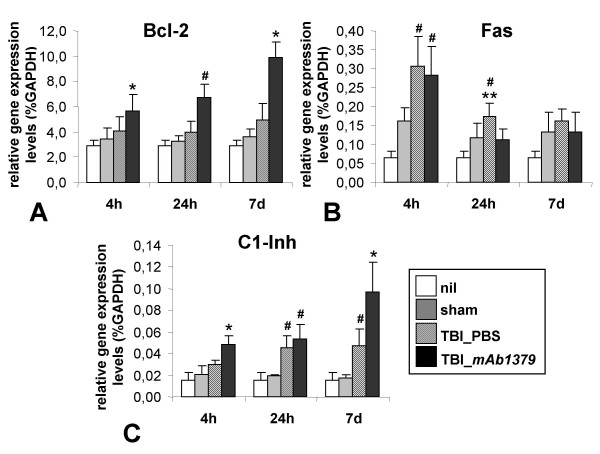
**Regulation of intracerebral expression of Bcl-2 (A), Fas (B), and C1-Inh (C) genes after head injury, as assessed by semi-quantitative two-step real-time RT-PCR**. Total RNA was extracted from homogenized murine brains at 4 hours, 24 hours, and 7 days after traumatic brain injury (TBI). The murine primers for GAPDH, Bcl-2, Fas and C1-Inh are depicted in table 1. The technique for real-time RT-PCR analysis is described in the methods section. See text for details. Data are shown as means ± SD of *n *= 3 in the "nil" group and *n *= 6 per time-point in all other groups. *P < 0.05 for TBI_*mAb 1379 *vs. TBI_placebo groups; ^#^*P *< 0.05 for TBI *vs*. sham groups; **P < 0.05 for TBI_placebo vs. TBI_*mAb 1379 *vs. placebo group (unpaired Student's *t*-test).

Expression of intracerebral genes of interest was assessed by semi-quantitative real-time RT-PCR analysis of brain tissue homogenates using mouse-specific primers (table 1). These included each a pro-apoptotic (Fas) and anti-apoptotic (Bcl-2) gene and a representative complement regulatory gene of the classical pathway (Inh). The baseline expression of these candidate genes was determined in brain homogenates from untreated normal mice („nil“ group, *n *= 3 per gene, Fig. 7). Sham-operated control mice (*n *= 6 per gene and time-point) showed a non-significant increase in the expression of Bcl-2, C1-Inh, and Fas at each time point assessed („sham“ group, *n *= 6 per gene and time-point, Fig. 7).

After head trauma, the *mAb1379*-injected mice showed a significant upregulation of the protective Bcl-2 and C1-Inh genes for up to 7 days, as compared to placebo-injected or sham-operated mice (*P *< 0.05, unpaired Student's *t*-test; *n *= 6 per gene, time-point, and TBI group Fig. 7). In contrast, Fas gene expression in injured brains showed different kinetics of regulation, with mRNA levels being significantly elevated in both TBI groups (placebo and *mAB 1379*) as early as 4 hours after trauma, compared to sham-operated mice (*P *< 0.05, unpaired Student's *t*-test; *n *= 6 per gene, time-point, and TBI group Fig. 7). As opposed to the Bcl-2 and C1-Inh genes, no significant differences in Fas gene expression were seen between the *mAB 1379 *and placebo-control groups at 4 hours and 7 days after trauma (Fig. 7). However, at 24 hours, Fas mRNA levels were significantly suppressed in the treatment group, reaching similar low levels as the sham controls (*P *< 0.05, *mAb1379 vs*. PBS group; Fig. 7).

## Discussion

Therapeutic modalities for inhibition of the complement cascade have been assessed in different models of brain injury in the past [5,17,23]. Most of these studies have used pharmacological approaches which led to complete "shut-down" of the complement system at the level where the three different activation pathways merge by inhibiting the C3 convertases (Fig. 1) [20,21,25,26]. A recent experimental study from our laboratory suggests, however, that the alternative pathway may be of particular importance in mediating neuroinflammation and neuronal cell death after head injury, based on studies in factor B gene knockout (*fB-/-*) mice [35]. The selective inhibition of the alternative pathway only has received increasing attention in various inflammatory diseases outside the CNS, due to recent findings which support its essential role in contributing to secondary tissue injury [31,32]. Based on our recent findings of a significant neuroprotection in *fB-/- *mice after TBI, we sought to extrapolate these findings to a pharmacological model by targeted inhibition of the alternative pathway [35]. We therefore used a newly available, highly specific and potent inhibitor of the alternative complement pathway, the *mAb 1379 *monoclonal anti-factor B antibody, in the identical head injury model. This antibody was previously shown to protect form inflammation and severity of disease in allergic airway inflammation, renal ischemia/reperfusion syndrome, and anti-phospholipid antibody-induced pregnancy loss in mice [34,36,37].

In the present study, we randomized adult male C57BL/6 to receive a systemic injection of either 1 mg *mAb 1379 *or placebo (vehicle only) at 1 hour and 24 hours after closed head injury. The selected dosage was in the titrated range used in previous studies on other murine models of inflammation [34,36,37]. The systemic (i.p.) route of administration and the time window of injection were selected based on the rationale that in this model system, the blood-brain barrier is breached as early as 1 hour after trauma, peaking at 4 hours, and persisting for up to 24 hours [38,41]. These kinetics of blood-brain barrier opening offer a "time window of opportunity" for peripherally administered compounds to reach the intrathecal compartment and exert pharmacological effects in the inflamed CNS, as previously shown for other pharmacological agents [26,39,40,42]. Furthermore, the post-trauma systemic injection within 1 to 24 hours after injury represents an approach with potential clinical implications [10,14].

Our data demonstrate that the *mAb 1379 *represents a potent complement inhibitor after TBI, based on a significant attenuation of alternative pathway complement activity (zymosan assay) and a significant inhibition of complement anaphylatoxin C5a levels (ELISA data) at 4 and 24 hours after trauma, compared to placebo controls. However, while the injection of 1 mg *mAb 1379 *induced a complement inhibition for up to 24 hours, the repeated injection at this time-point was obviously not sufficient for sustaining a prolonged inhibition of complement activation until 7 days after injury. In other experimental models of inflammation, we have recently found that the hepatic factor B synthesis is increased due to initiation of the acute-phase response, thus necessitating higher doses of *mAb 1379 *for complete inhibition (Holers VM, Thurman JM; *unpublished observations*).

Aside from the shortcoming of limited complement inhibition related to the half-life of the compound, compensatory inflammatory reactions may also account for the lack of neurological improvement. These compensatory effects include the release of pro-inflammatory cytokines in the injured brain, such as tumor necrosis factor (TNF) and of interleukins (IL) -1β, -8, -12, -18, and other mediators of neuroinflammation [2,45,46]. Finally, the neurological score used in the present study (NSS), albeit widely used with success in previous studies on this model system [26,27,39-43], may not be sensitive enough to detect subtle changes in performance attributed to morphological alterations of cerebral tissue damage. Thus, other neurological testing systems may have to be applied in future studies to test the relevance of this compound in neurotrauma in more detail, including the Morris water maze for assessment of memory tasks.

Despite the lack of neurological improvement in the *mAb 1379*-treated mice, we observed an impressive extent of neuroprotection at the tissue level and a significant induction of neuroprotective genes in the injured brain. Specifically, the *mAb 1379*-treated mice had an attenuated extent of neuronal cell death and a preserved cortical microarchitecture for up to 7 days after head injury, compared to placebo controls. These promising findings imply that with a modified protocol of *mAb 1379 *administration, e.g. by higher doses or repeated injections every 24 hours for the first week, may lead to an increased extent of cerebral neuroprotection which will likely influence the outcome at a clinical-neurological level. Another strategy could involve the use of therapies targeted to the brain using CR2-linked chimeras which might provide more complete local control of complement activation [47,48]. This hypothesis will have to be tested in future experimental studies.

## Conclusion

The alternative pathway of complement activation appears to play a more crucial role in the pathophysiology of complement-mediated neuroinflammation after TBI than previously appreciated. In the present study, we extrapolated previous findings of neuroprotection in factor B gene-deficient (*fB-/-*) mice [35] to a pharmacological approach using a specific and potent inhibitor of the alternative complement pathway (*mAb 1379*). The randomized treatment protocol used in this experimental study on closed head injury in mice revealed the following *mAb 1379*-mediated beneficial effects, as compared to placebo controls:

*(1) *A significant attenuation of complement pathway activity at the level of the alternative pathway (zymosan assay) and overall at the level of anaphylatoxin formation (C5a ELISA).

*(2) *An impressive reduction of neuronal cell death (TUNEL) and a restoration of cortical cell layers in the injured hemisphere (immunohistochemistry).

*(3) *A significant upregulation of candidate neuroprotective genes in the injured hemisphere (real-time RT-PCR).

However, these neuroprotective effects at the tissue level did not extend to an improved neurological outcome or to reduced mortality in *mAb 1379*-treated mice, as compared to placebo controls. The observation of elevated factor B levels in the intrathecal compartment of severely head-injured patients further supports the pharmacological concept of a specific inhibition of factor B [49]. However, prior to extrapolation to the clinical setting, further animal studies will be required for determining the optimal dosage and injection intervals in experimental models of head injury.

## Abbreviations

Central nervous system (CNS); diaminobenzidine (DAB); 4',6'-diamino-2-phenylindole (DAPI); enzyme-linked immunosorbent assay (ELISA); glial fibrillary acidic protein (GFAP); neuron-specific nuclear protein (NeuN); o-phenylenediamine dihydrochloride (OPD); phosphate-buffered saline (PBS); real-time reverse transcriptase polymerase chain reaction (real-time RT-PCR); room temperature (RT); sodium dodecyl sulfate-polyacrylamide gel electrophoresis (SDS-PAGE); traumatic brain injury (TBI); terminal deoxynucleotidyl transferase (TdT); terminal deoxynucleotidyl transferase biotin-dUTP nick end labeling (TUNEL).

## Competing interests

Dr. Holers receives consultation fees and stock from Taligen Therapeutics, which has licensed complement-based technology currently submitted for patent from the University of Colorado at Denver and Health Sciences Center. Drs. Thurman and Stahel are co-applicants on this patent. There are no other conflicting financial interests by any of the authors regarding the present project.

## Authors' contributions

IL, OIS, WE, and PFS were responsible for conception and planning of the experiments, performing of all animal experiments, analysis of the data and writing of the manuscript. VMH and JMT provided the anti-factor B antibody and performed the zymosan assay. IL, AMH, MET, and MR performed the TUNEL and immunohistochemistry experiments. IL, MR and DH performed the real-time RT-PCR analyses. DR and PAW performed the murine C5a ELISA experiments. All authors read and approved the final manuscript.
